# Myositis/Myasthenia after Pembrolizumab in a Bladder Cancer Patient with an Autoimmunity-Associated HLA: Immune–Biological Evaluation and Case Report

**DOI:** 10.3390/ijms22126246

**Published:** 2021-06-10

**Authors:** Cirino Botta, Rita Maria Agostino, Vincenzo Dattola, Vittoria Cianci, Natale Daniele Calandruccio, Giovanna Bianco, Antonino Mafodda, Roberto Maisano, Eleonora Iuliano, Giovanna Orizzonte, Domenico Mazzacuva, Antonia Consuelo Falzea, Rita Emilena Saladino, Rocco Giannicola, Giorgio Restifo, Umberto Aguglia, Michele Caraglia, Pierpaolo Correale

**Affiliations:** 1 Unit of Hematology, Azienda Ospedaliera “Annunziata”, 87100 Cosenza, Italy; 2 Hematology Unit, Department of Health Promotion, Mother and Child Care, Internal Medicine and Medical Specialties (PROMISE), University of Palermo, 90128 Palermo, Italy; 3 Unit of Oncology, Unit. Grand Metropolitan Hospital “Bianchi Melacrino Morelli”, 89124 Reggio Calabria, Italy; ritamariaagostino@yahoo.it (R.M.A.); danielecala@hotmail.com (N.D.C.); bianco.giovanna_@libero.it (G.B.); antoninomafodda@gmail.com (A.M.); robertomaisano@alice.it (R.M.); eleonorafiuliano@hotmail.it (E.I.); g.orizzonte@aorc.it (G.O.); antonellafalzea@gmail.com (A.C.F.); roccogiannicola@gmail.com (R.G.); 4 Unit of Neurology, Unit. Grand Metropolitan Hospital “Bianchi Melacrino Morelli”, 89124 Reggio Calabria, Italy; vincenzodattola@gmail.com (V.D.); vittoriacianci@virgilio.it (V.C.); u.aguglia@unicz.it (U.A.); 5 Laboratory of Autoimmunity, Unit. Grand Metropolitan Hospital “Bianchi Melacrino Morelli”, 89124 Reggio Calabria, Italy; domenico.mazzacuva@aorc.it; 6 HLA Tissue Typing Laboratory, Unit. Grand Metropolitan Hospital “Bianchi Melacrino Morelli”, 89124 Reggio Calabria, Italy; ritaemilena.saladino@gmail.com; 7 Nuclear Medicine Unit, Grand Metropolitan Hospital “Bianchi Melacrino Morelli”, 89124 Reggio Calabria, Italy; giorgio.restifo@aorc.it; 8 Department of Precision Medicine, University of Campania “L. Vanvitelli”, 80138 Naples, Italy; michele.caraglia@unicampania.it; 9 Laboratory of Precision and Molecular Oncology, Biogem Scarl, Institute of Genetic Research, 83031 Ariano Irpino, Italy

**Keywords:** urothelial cancer, PD1-checkpoint inhibitors, class-I/II HLA, myasthenia

## Abstract

Pembrolizumab (mAb to PD-1) has been recently approved for the therapy of pretreated urothelial cancer. Despite the efficacy, it is often accompanied by unpredictable and sometime severe immune-related (ir) adverse events (AEs). Here, we report the clinical and immune–biological characterization of a patient with a metastatic bladder cancer who developed myositis signs (M) and a myasthenia-like syndrome (MLS) during treatment with pembrolizumab. The patient presented an autoimmunity-associated HLA haplotype (HLA-A*02/HLA-B*08/HLA-C*07/HLA-DRB1*03) and experienced an increase in activated CD8 T-cells along the treatment. The symptomatology regressed after pembrolizumab discontinuation and a pyridostigmine and steroids-based therapy. This is the first report of concurrent M and MLS appearance in cancer patients receiving pembrolizumab. More efforts are needed to define early the risk and the clinical meaning of irAEs in this setting.

## 1. Introduction

Tumor-infiltrating T-cell rescue by using PD-1/PDL1 immune checkpoint-blocking mAbs represents an innovative therapeutic strategy for a number of different malignancies, including NSCLC, malignant melanoma and urothelial, as well as head and neck cancer [1,2,3]. Although very active in a number of patients in terms of clinical benefit and survival, this treatment could be complicated by severe immune-related (ir) adverse events (AEs) (irAEs) [4]. Interestingly, these immunological events have been occasionally reported to correlate with a good outcome in different malignancies including malignant melanoma, colorectal cancer and mNSCLC [5,6,7,8]. However, less is known for patients with urological malignancies, where the study of potential correlations between outcome and irAEs under immune-checkpoint inhibitors treatment is still in its infancy (it has been recently demonstrated that patients responding to anti-PD-1/L1 treatment are more likely to develop irAEs) [9].

It has been hypothesized that these adverse events are strictly related to the immunological nature of the treatment that, other than impairing tolerance against cancer (private) antigens, even breaks endogenous tolerance to multiple and different (physiological) self-antigens [4,10,11]. At present, however, no clear classification of tumor-specific irAEs has been proposed, thus the report of unexpected and infrequent irAEs is eagerly welcome.

## 2. Case Report

**Figure 1:**
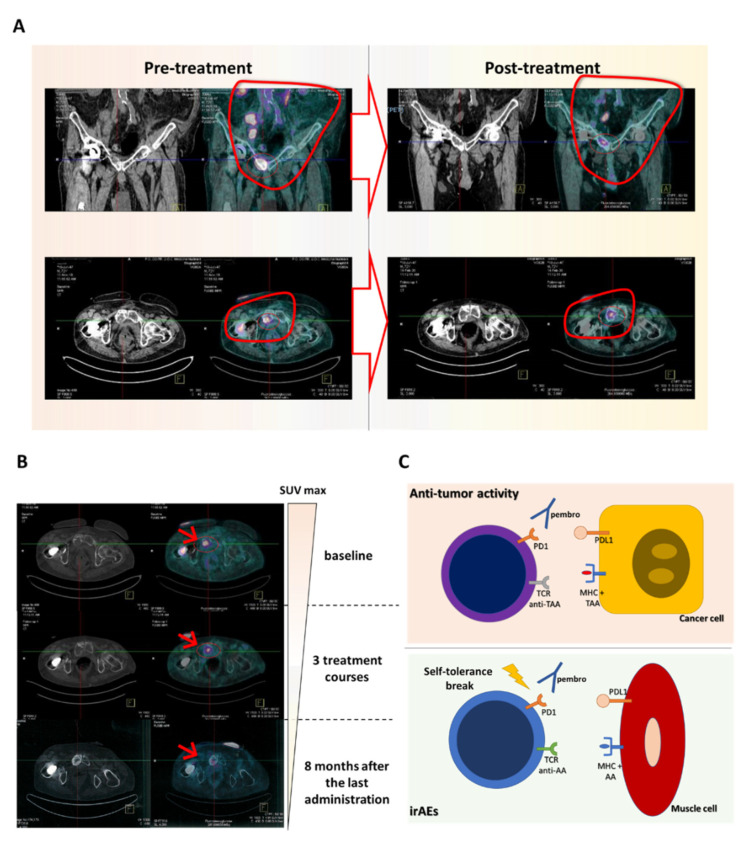
(**A**) CT/PET scan performed prior to and after three treatment cycles of pembrolizumab showed a good disease control with volume reduction in all the involved sites. (**B**) CT/PET scan performed during the follow-up (after 8 months from the last pembrolizumab administration) demonstrated a further reduction in cancer metabolism coupled with a sclerotic reaction. Arrows indicate the point where SUV reduction is more evident. (**C**) Cartoon representing the possible mechanism responsible for (muscle) immune-related adverse events (irAEs). Pembro: pembrolizumab; AA: auto-antigens; TAA: tumor-associated antigens.

VG is a seventy-two year-old male with a good performance status, with a moderate smoking habit, no professional or familiar risk of bladder malignancy or autoimmunity and hypertension in medical treatment, who was diagnosed with a locally advanced bladder cancer for which he underwent radical cystectomy and locoregional lymphadenectomy on October 2018. Histological examination detected an undifferentiated urothelial carcinoma infiltrating the muscularis mucosae with no nodal involvement. A pre-operatory radiological study excluded the presence of disseminated disease. He was therefore staged as T3N0Mo and no adjuvant chemotherapy was recommended. On February 2019 he become symptomatic with a rise in sacral and pelvic pain associated with weight loss and ankle swelling. A CT/PET scan detected the presence of soft tissue relapse within the left ileo-psoas muscle as well as bone (costs, pelvis, ischiopubic bones) and iliac, lombo-aortic and inguinal node metastasis. This patient was therefore addressed to palliative chemotherapy with cisplatin and gemcitabine that was ineffective in controlling the disease and was suspended just after three cycles upon instrumental demonstration of disease progression (from 20 March to 10 May 2019). Subsequently, he received radiation therapy (8Gy) and then metronomic chemotherapy with oral vinorelbine (20 mg three times a week) from 1 September to 10 December 2019, before new disease progression was demonstrated (Figure 1A). He therefore was addressed to immunotherapy, receiving pembrolizumab at a dose of 200 mg every three weeks, starting on 17 December 2019. He showed a fast benefit in terms of quality of life and pain decrease and swelling after the first administration. The treatment was well tolerated and he could receive three treatment cycles with no alterations in blood tests or symptoms suggestive for irAEs, with the exception of a mild arthromyalgia (g1), stiffness and moderate asthenia. A few days after the third treatment course he developed left eyelid ptosis, which progressively worsened until becoming bilateral within a few days. In parallel, diplopia with limitation of eye movements, proximal weakness to the limbs and dysphonia also occurred. A biochemical blood test showed no change in inflammatory markers and blood cell counts, a sudden rise in cell lysis enzymes and increased levels of anti-nuclear antibodies (ANA) (Table 1).

The study of his peripheral blood mononuclear cells performed by flow cytometry revealed a decline in CD4^+^/CD8^+^ T-cell ratio and a rise in activated T cells (Table 1).

As long as these symptoms were rising, this patient was hospitalized. Contrast-enhanced brain MRI, hearth ultrasonography, single-fiber electromyography and repetitive nerve stimulation test, lumbar puncture and subsequent cerebrospinal fluid analysis were carried out (Table 2) in order to exclude possible metastases, traumas or infectious diseases. We then performed laboratory research of M- and MSL-specific immunoglobulins, detecting the presence of AAbs to the muscle acetylcholine receptors (AChR-abs) (while AAbs to muscle-specific kinase (MuSK) remained within the normal ranges) commonly associated with myasthenia. A HLA genotype study of this patient finally confirmed the expression of a high-risk autoimmunity haplotype (Table 2). Additionally, this finding was accompanied by a reduction in the CD4/CD8 ratio and an increase in the presence of activated HLADR+ cells.

The patient discontinued immunotherapy and received a proper treatment, including intravenous dexamethasone (16 mg a day) associated with oral pyridostigmine bromide (60 mg three times a day), that led to full recovery of the patient within fifteen days and hospital discharge in a good clinical shape. The patient was re-evaluated by a CT/PET scan, showing a partial antitumor response (Figure 1A). He remained free of symptoms for a further six months, showing no blood cell, instrumental or biochemical alterations (Table 1). He continued the treatment with oral prednisone (10 mg a day) and pyridostigmine bromide until July 2020 when a distal urethral soft tissue relapse required palliative radiation therapy (30Gy in ten fraction). Our patient is currently in a good clinical shape, free from systemic progression with no further neurologic impairment or other irAEs or signs of other common adverse events, sixteen months after the last pembrolizumab dose on February 2020 (Figure 1B).

## 3. Discussion

PD1/PDL1 immune-checkpoint blockade is often complicated by irAEs whose occurrence is unpredictable and heterogenous in term of physiopathology, symptoms and severity. The majority of them usually present mild (g1–2 irAEs) articular, endocrine, mucosal and cutaneous symptoms, while 10–14% develop more severe irAEs (g3–4), including pneumonia, hypophysitis and neurological impairments [4,11,12,13]. It is reasonable to believe that irAEs present substantial differences correlated with gender, class I/II HLA-typing, malignant histology and metastatic sites, as well as with the different PD-1/PDL1 mAb (used alone or in a combination strategy) [14,15,16,17]. All these factors may affect the release and processing of different tissue-specific auto-antigens and relative immune-inflammatory response, sufficient to ignite an autoimmune response. Among them, idiopathic inflammatory myopathies represent rare autoimmune diseases defined by muscle weakness and heterogeneous systemic organic involvement. Interestingly, a irAEs involving muscles have been reported to occur in cancer patients receiving immune-checkpoint blockade in a variable range of 1–43% [4,18,19,20,21,22,23,24]. Additionally, several fatal events (especially if associated with heart muscle damage) have been reported [25].

Tumor microenvironment is characterized by an unstable equilibrium where cells belonging to different arms of the immune system (including lymphocytes and myeloid-derived cells) create a tolerogenic niche where malignant cells could freely proliferate [26,27,28,29,30,31,32,33,34]. This aspect closely resembles pro-inflammatory/tolerogenic phenomena happening around an injured tissue, a mechanism which physiologically protects us from the development of autoimmune diseases. Accordingly, the development of irAEs after PD1/PDL1 inhibition could indeed rely (from a pathogenetic point of view) on mechanisms of “epitope-sharing” between normal and cancer cells or “epitope-spreading” (release in the microenvironment of self-antigens as a consequence of tumor cell death) in the cross-activation of T cells against health tissues [24], which finally cause an inappropriate immune-mediated inflammatory response to muscle cells (Figure 1C). This point, in particular, warrants further investigation; in the view of a broader context of “private” tumor-associated antigens which could trigger irAEs, the advent of whole exome sequencing methodologies and computational docking strategies, able to derive the final 3D structure of the HLA–epitope association, will clarify these aspects in the future [35].

Additionally, the specific rise in AABs and specific genetic risk partially related to class I and II HLA should been taken carefully into account [15,36,37]. Indeed, the expression of HLA-B*08 as well as DRB1*03, *07 or *11 are all correlated with the development of myositis in not-neoplastic patients [38]. Specifically, the risk of early onset myasthenia has been correlated with the presence of HLA-A*01 and *02 together with B*08, C*05 and *07 and DRB1*03, *07 and *13 [37].

On the other hand, acquired myasthenia gravis is another rare antibody-mediated autoimmune disease caused by impaired neuromuscular transmission that leads to abnormal muscle fatigability, affecting in some cases only the eye muscles (ocular MG), but in most cases several muscle groups. It often occurs as para-neoplastic syndromes associated with thymoma, endocrine neoplasms and malignant melanoma [39,40]. The etiology seems to be very complex and includes both environmental and genetic risk factors mainly involving (again) Class I and II HLA complexes. Indeed, the risk of early onset myasthenia has been correlated with the germinal expression of HLA-A*01 and *02 together with B*08, C*05 and *07 and DRB1*03, *07 and *13 [37]. The occurrence of symptoms in these patients is strictly correlated with the presence of AChR-abs (80–85% of the cases) and/or to MuSK AAbs (10–50%) [41,42]. The prognosis of the disease depends on the possibility to treat the “triggering” malignancy as well as on the response to therapeutics such as steroids, immunoglobulins or plasmapheresis. However, a recent pooled analysis suggested an overall dismal outcome for these patients [12,43,44]. The occurrence of myasthenia with myositis signs has been sporadically observed in patients with malignant melanoma, NSCLC and kidney cancer receiving PD-1/PDL1 blockade but, so far, it has never been recorded in patients with urothelial cancer.

In our patient, the hypothesis that both myositis- and myasthenia-like syndrome are consequent to pembrolizumab treatment is sustained by clinical as well as biological/biochemical considerations. In the first place, the symptoms occurred a few months after the beginning of the treatment in concomitance with a significant anti-tumor response, progressive rise in muscular cell lysis markers such as ALT, AST, CK, CK, MB and LDH, a significant serum conversion for ANA, anti-thyroglobulin Abs and finally, AChR-abs that confirmed the diagnosis of myasthenia. Additionally, all symptoms disappeared upon corticosteroid treatment and pembrolizumab discontinuation excluding the hypothesis of a paraneoplastic condition. Our analysis of his HLA genotype revealed the presence of an autoimmune haplotype presenting class I HLA alleles associated with an increased risk of both myositis (A*02, B*08, C*07, DRB1*03) and myasthenia gravis (B*08 and DRB1*03) [36,37].

## 4. Conclusions

The fact that irAEs symptoms occurred in parallel with an increased serum level of AAbs and clinical response to pembrolizumab suggests that the equilibrium between antitumor activity and tolerance to auto-antigens was affected by the immunological treatment. On the basis of these considerations, we believe that the research of autoimmune-associated HLA haplotypes prior starting PD-1/PDL1 immune-checkpoint mAb administration could be a safe modality to warn physicians of an increased risk of life-threatening irAEs.

## Figures and Tables

**Table 1:** Immune and inflammatory parameters modulation along pembrolizumab treatment.

Blood Tests	Baseline	Post-Treatment	Follow-Up
**Inflammatory markers**			
**CRP (mg/L)**	40.7 ^(#)^	15.5 ^(#)^	6.59 ^(#)^
**ESR (mm/h)**	104 ^(#)^	50 ^(#)^	24 ^(#)^
Cell lysis enzymes			
AST (U/L)	35	445 ^(#)^	19
ALT (U/L)	15	109 ^(#)^	7
LDH (U/L)	154	4403 ^(#)^	177
CK (U/L)	ND	4403 ^(#)^	100
CK MB(U/L)	ND	189 ^(#)^	9
Troponin I (ng/mL)	ND	5.42 ^(#)^	<0.012
Auto-antibodies			
ASMA	ND	negative	negative
ENA	ND	negative	negative
ANA	ND	1/1280 ^(#)^	1/160 ^(#)^
Anti-peroxidase (U/l)	ND	94.64 ^(#)^	125.80 ^(#)^
Anti-ChR-abs (U/mL)	ND	2.8 ^(#)^	2.68 ^(#)^
Anti-MuSK (U/mL)	ND	<0.4	<0.4
**CD4+/CD8+ T cell ratio**	1.50	1.1	ND
**Activated T cells % (HLA-DR+)**	21	27	ND
**Activated T cells % (HLA-DR+)**			

# out of normality range.

**Table 2:** Instrumental evaluation and HLA determination at adverse event onset.

Patient’s Characteristics	
Contrast-enhanced brain MRI	No CNS metastases
Single-fiber EMG and repetitive nerve stimulation test	Within normal ranges
Cerebrospinal fluid	Within normal ranges
hearth ultrasonography	Within normal ranges
HLA haplotype	HLA-A*02/*29 HLA-B*08/*35 HLA-C*04/*07 DRB1*03/*04

## Data Availability

The data presented in this study are available on request from the corresponding author.
